# SARS-CoV-2 Infection Modulates ACE2 Function and Subsequent Inflammatory Responses in Swabs and Plasma of COVID-19 Patients

**DOI:** 10.3390/v13091715

**Published:** 2021-08-28

**Authors:** Lucía Gutiérrez-Chamorro, Eva Riveira-Muñoz, Clara Barrios, Vanesa Palau, Maria Nevot, Sònia Pedreño-López, Jordi Senserrich, Marta Massanella, Bonaventura Clotet, Cecilia Cabrera, Oriol Mitjà, Marta Crespo, Julio Pascual, Marta Riera, Ester Ballana

**Affiliations:** 1 IrsiCaixa-AIDS Research Institute, Health Research Institute Germans Trias i Pujol (IGTP), Universitat Autònoma de Barcelona, 08916 Badalona, Spain; lgutierrez@irsicaixa.es (L.G.-C.); eriveira@irsicaixa.es (E.R.-M.); mnevot@irsicaixa.es (M.N.); spedreno@irsicaixa.es (S.P.-L.); jsenserrich@irsicaixa.es (J.S.); mmassanella@irsicaixa.es (M.M.); bclotet@irsicaixa.es (B.C.); ccabrera@irsicaixa.es (C.C.); 2 Hospital del Mar Department of Nephrology, Hospital del Mar Medical Research Institute (IMIM), 08003 Barcelona, Spain; cbarrios@psmar.cat (C.B.); vpalau@imim.es (V.P.); mcrespo@psmar.cat (M.C.); jpascual@psmar.cat (J.P.); 3 Fight AIDS and Infectious Diseases Foundation, 08916 Badalona, Spain; omitja@flsida.org; 4 Hospital Universitari Germans Trias i Pujol, 08916 Badalona, Spain; 5 Universitat Central de Catalunya, Universitat de Vic, 08500 Vic, Spain; 6 Lihir Medical Centre-International SOS, Londolovit, Lihir Island, Papua New Guinea

**Keywords:** SARS-CoV-2-ACE2, inflammation, biomarker

## Abstract

Angiotensin converting enzyme 2 (ACE2) is a host ectopeptidase and the receptor for the SARS-CoV-2 virus, albeit virus-ACE2 interaction goes far beyond viral entry into target cells. Controversial data exists linking viral infection to changes in ACE2 expression and function, which might influence the subsequent induction of an inflammatory response. Here, we tested the significance of soluble ACE2 enzymatic activity longitudinally in nasopharyngeal swabs and plasma samples of SARS-CoV-2 infected patients, along with the induction of inflammatory cytokines. Release of soluble functional ACE2 increases upon SARS-CoV-2 infection in swabs and plasma of infected patients, albeit rapidly decreasing during infection course in parallel with *ACE2* gene expression. Similarly, SARS-CoV-2 infection also induced the expression of inflammatory cytokines. These changes positively correlated with the viral load. Overall, our results demonstrate the existence of mechanisms by which SARS-CoV-2 modulates ACE2 expression and function, intracellular viral sensing and subsequent inflammatory response, offering new insights into ACE2 dynamics in the human upper respiratory tract and pointing towards soluble ACE2 levels as a putative early biomarker of infection severity.

## 1. Introduction

Angiotensin-converting enzyme 2 (ACE2) is the cellular receptor for severe acute respiratory syndrome coronavirus 2 (SARS-CoV-2), the etiological agent of the COVID-19 disease [1]. SARS-CoV-2 infection is manifested in a wide range of symptoms [2,3], with the risk for increased disease severity correlating with male gender, advanced age and underlying comorbidities [4,5].

The consequences of SARS-CoV-2 interactions with ACE2 are controversial. ACE2 is known to be a tissue-damage protective factor, due to its monocarboxipeptidase actions on Angiotensin (ANG)-II promoting anti-inflammatory functions through Ang-(1-7) production. These two peptides of the renin angiotensin system (RAS) are involved in the regulation of blood pressure and body fluid homeostasis [6,7,8]. ACE2 ectodomain is released from the cell surface by ADAM17 and TMPRSS2 carboxipeptidases [9,10], TMPRSS2 being also necessary for SARS-CoV-2 viral entry due to its function in the priming of viral S protein [9]. Interestingly, once released, the ectodomain of ACE2 keeps the catalytic activity which is able to cleave circulating vasoactive peptides [7,11] and, in the context of SARS-CoV-2 infection, might also significantly influence viral spread and pathogenesis, either by limiting new viral infections acting as a soluble decoy or, alternatively, limiting local inflammation due to its tissue protective function. In this sense, elevated circulating ACE2 levels have been reported in different cohorts of COVID-19 patients, suggesting a relation between clinical course and ACE2 levels either in acute or long-term disease [12,13,14,15], although data needs further validation.

One of the hallmarks of COVID-19 is a dysregulated antiviral immune response [16,17]. Interestingly, ACE2 expression is reported to be induced upon exposure to an interferon (IFN) [18,19] following SARS-CoV-2 infection, in contrast with data from the SARS-CoV virus, where cellular ACE2 expression levels are downregulated by the viral infection and linked with the pathogenicity of the virus [20]. It has also been proposed that SARS-CoV-2 may antagonize initial viral detection and IFN responses, although the mechanism is still unknown [21,22] and the temporal relationship between viral load and host gene expression has not been explored in vivo in patients. At gene transcription level, shotgun RNA sequencing profiles of nasopharyngeal samples from infected subjects associated IFN stimulated gene (ISGs) expression with viral load, age and sex [23]. In this context, the in-depth analysis of nasopharyngeal samples may help to decipher the intricate processes of SARS-CoV-2 infection and the host immune response. Gathering this information is crucial to better understand how the initial phase of infection may compromise the disease progression.

Thus, studies focused on key molecular mechanisms early after SARS-CoV-2 infection are of great value for better deciphering the inflammation cascade and immune signaling in the initial phase of the disease. Moreover, the role of IFN in ACE2 expression and shedding should be deeply evaluated because of the important implications in modulating inflammatory cytokines. Here, we evaluated the interplay between ACE2 function and host immune response in nasopharyngeal samples and plasma of a cohort of SARS-CoV-2 infected subjects, delineating how viral infection modulates both ACE2 expression and function in vivo, as well as its relation with immune response and viral load.

## 2. Materials and Methods

### 2.1. Patients and Samples

Nasopharyngeal swabs were obtained from 40 patients recruited under the BCN PEP CoV-2 Study (NCT04304053) during the spring 2020 SARS-CoV-2 outbreak in Catalonia (north-east Spain). All COVID-19 cases included in the present analysis were adults (i.e., ≥18 years of age) with quantitative PCR result available at baseline and symptoms that met the diagnostic criteria for COVID-19 (fever, acute cough, sudden olfactory or gustatory loss or rhinitis). Two nasopharyngeal swabs were obtained at the time of recruitment (day 0) and at follow-up, 3 days after (day 3). Full details of the original study are reported elsewhere [24]. Similarly, a group of COVID-19 negative nasopharyngeal swabs (*n* = 20) were collected following the same procedure and matched by age and sex to the infected patients (mean age: 39 years and 70% females). All negative samples were tested for SARS-CoV-2 infection by PCR with a negative result. Plasma samples were obtained from 75 acute infected patients attended at the COVID-19 unit of Hospital Germans Trias i Pujol. When possible, patients were longitudinally followed for a median of 27 days after first visit. The study protocol was approved by the institutional review board of Hospital Germans Trias i Pujol. All participants provided written informed consent.

### 2.2. ACE2 Enzymatic Assay

The ACE2 fluorescent enzymatic assay protocol was performed as previously described [25], using an ACE2-quenched fluorescent substrate (Mca-Ala-Pro-Lys(Dnp)-OH; Enzo Life Sciences, Lausen, Switzerland). Swab samples (5 μL) were incubated with ACE2 assay buffer (100 mM Tris·HCl, 600 mM NaCl and 10 μM ZnCl_2_, pH 7.5 in presence of protease inhibitors 100 μM captopril, 5 μM amastatin, 5 μM bestatin and 10μM Z-Pro-prolinal (from Merck-Sigma-Aldrich and Enzo Life Sciences, Madrid, Spain and Lausen, Switzerland) and 10 μM fluorogenic substrate in a final volume of 100 μL at 37 °C for 18 h. Plasma samples (2 μL) were also incubated with the same ACE2 assay buffer plus 0.5 mM ZnCl_2_ as previously published [26]. To test substrate specificity, an ACE2-specific inhibitor, MLN-4760 (Merck-Sigma-Aldrich, Darmstadt, Germany), was added. Soluble ACE2 cleaves the substrate proportionally to the enzyme activity. Measurements were carried out in duplicate for each data point. Plates (Corning clear flat-bottom 96 well black polystyrene plates) were read using a fluorescence plate reader (Ensight^TM^ multimode plate reader, Perkin Elmer, Waltham, USA) at λ_ex_320 nm and λ_em_400 nm. Results were expressed as RFU (relative fluorescent units)/µL sample/h.

### 2.3. SARS-CoV-2 PCR Detection and Viral Load Quantification

SARS-CoV-2 viral load was quantified from nasopharyngeal swabs at the distinct time points by quantitative reverse transcription PCR (qRT-PCR). RNA extraction was performed using MagMAX™ Pathogen RNA/DNA Kit (4462359, Fisher Scientific, Vilnius, Lithuania), optimized for a KingFisher instrument (ThermoFisher, Vantaa, Finland), following manufacturer’s instructions. PCR amplification was based on the 2019-Novel Coronavirus Real-Time RT-PCR Diagnostic Panel guidelines and protocol developed by the American Center for Disease control and Prevention. Briefly, a 20 μL PCR reaction was set up containing 5 μL of RNA, 1.5 μL of N_2_ primers and probe (2019-nCov CDC EUA Kit, #10006770, Integrated DNA Technologies, Coralville, IA, USA) and 12.5 μL of TaqPath 1-StepRT-qPCR Master Mix (ThermoFisher, Frederick, MD, USA). Thermal cycling was performed at 50 °C for 15 min for reverse transcription, followed by 95 °C for 2 min and then 45 cycles of 95 °C for 3 s and 55 °C for 30 s in the Applied Biosystems 7500 or QuantStudio5 Real-Time PCR instruments (ThermoFisher, Foster City, CA, USA). For absolute quantification, a standard curve was built using 1/5 serial dilutions of a SARS-CoV-2 plasmid (2019-nCoV_N_Positive Control, #10006625, 200 copies/μL, Integrated DNA Technologies, Coralville IA, USA) and run in parallel in all PCR determinations. Viral load of each sample was determined in triplicate and extrapolated from the standard curve, and corrected by the corresponding dilution factor.

### 2.4. Quantitative RT-Polymerase Chain Reaction (qRT-PCR)

RNA extraction was performed using a MagMAX™ Pathogen RNA/DNA Kit, optimized for a KingFisher instrument (ThermoFisher, Vantaa, Finland). Reverse transcriptase was performed using the TaqPath 1-StepRT-qPCR Master Mix (ThermoFisher, Frederick, MD, USA). mRNA relative levels of all genes were measured by two-step quantitative RT-PCR and normalized to GAPDH using the 1/DCt method. All probes were purchased from Life Technologies (TaqMan gene expression assays, Marsiling, Singapore).

### 2.5. IL6 Plasma Levels

Frozen plasma samples were analyzed for IL-6 using the human Magnetic Luminex Performance Assay (R&D Systems, Minneapolis, MN, USA). A 5-parameter standard curve was used to interpolate cytokine concentrations.

### 2.6. Statistical Analysis

Given that the normality was not fulfilled by the majority of variables, non-parametric methods were applied. Between group differences were evaluated through a Mann–Whitney U test. Wilcoxon signed-rank test was used to evaluate statistically significant changes in paired samples within the SARS-CoV-2 positive cohort (baseline-3 days). Correlations between variables were evaluated through Spearman’s rank correlation coefficients. Results were considered as statistically significant at *p*-value < 0.05. Statistical analysis was performed using STATA 15.1.

## 3. Results

### 3.1. SARS-CoV-2 Infection Modulates ACE2 Function and Expression in Upper Respiratory Tract

Because of the central role of ACE2 receptor as the viral entry point, deciphering the ACE2 functional role is critical for the understanding of the pathophysiological changes due to SARS-CoV-2 infection. Indeed, SARS-CoV-2 viral entry might trigger cleavage of the ACE2 receptor ectodomain, affecting ACE2 function and systemic release.

To gain insight into ACE2 function in viral entry and early infection events, we standardized the measurement of soluble ACE2 enzymatic activity in nasopharyngeal swabs as a surrogate marker of ACE2 function in the upper respiratory tract (Supplementary Figure S1). We observed significant ACE2 enzymatic activity levels in nasopharyngeal swabs, suggesting a potential role of ACE2 activity in the upper respiratory tract in SARS-CoV-2 pathogenesis.

**Figure 1:**
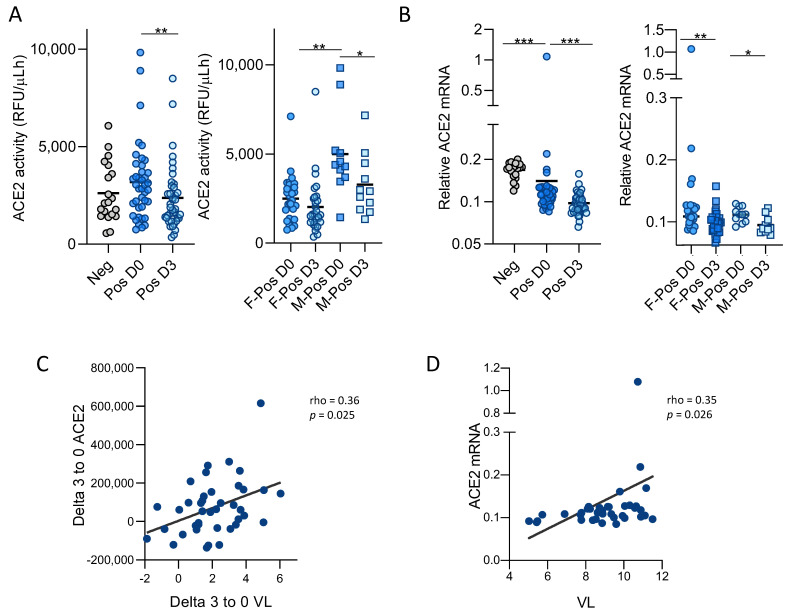
Enzymatic activity and expression of ACE2 in nasopharyngeal swabs from SARS-CoV-2 patients. (**A**) ACE2 enzymatic activity in nasopharyngeal swabs from negative and SARS-CoV-2 positive subjects. Left, *ACE2* levels in negative (*n* = 20, grey circles) or positive SARS-CoV-2 samples (*n* = 40), collected at the time of PCR positivity (D0, dark blue circles) and 3 days later (D3, light blue circles). Right, ACE2 activity in positive SARS-CoV-2 females (dark blue circles, F-Pos) and males (dark blue squares, M-Pos) collected at the time of PCR positivity (D0) and 3 days later (D3). Data are expressed as individual relative fluorescence units (RFU) per microliter/hour. (**B**) *ACE2* expression in nasopharyngeal swabs. Left, relative mRNA expression of *ACE2* gene in negative (*n* = 20, grey circles) or positive SARS-CoV2 samples (*n* = 40) at the time of PCR positivity (D0, dark blue circles) and 3 days later (D3, light blue circles). Right, relative expression of *ACE2* in positive SARSCoV2 subjects stratified by gender (F-Pos, female; M-Pos, male). Correlation of ACE2 activity and SARS-CoV-2 viral load overtime. (**C**) The decrease in viral load in 3 days positively correlated with the decrease of soluble ACE2 enzymatic activity. (**D**) Viral load positively correlated with *ACE2* gene expression Data are shown as mean ± SEM and analyzed by Mann-Whitney U test, * *p* < 0.05, ** *p* < 0.01, *** *p* < 0.001. Linear correlation (Spearman) r and *p*-values are shown.

Thus, soluble ACE2 activity was measured in a cohort of acute infected SARS-CoV-2 subjects, confirmed by a positive PCR diagnostic test, and in a group of uninfected controls, matched by age and sex. Epidemiological and clinical characteristics of the subjects included in the study are summarized in Table 1. SARS-CoV-2 positive subjects were longitudinally followed with a first sample at the time of the recruitment (day 0) and a second nasopharyngeal swab 3 days later. ACE2 activity slightly increased in SARS-CoV-2 positive samples upon infection (Figure 1A). Interestingly, ACE2 activity at day 3 was significantly lower than the first sample (Figure 1A), supporting the idea that ACE2 cleavage and release occurs upon viral entry and its function is not recovered thereafter.

When samples were stratified according to sex, ACE2 activity levels were higher in SARS-CoV-2 positive males compared to females, irrespective of the day of sampling (Figure 1A).

We next explored the temporal relationship of *ACE2* gene expression in nasopharyngeal swabs from our cohort of positive and negative SARS-CoV-2- subjects. Surprisingly, *ACE2* gene expression was significantly lower in positive SARS-CoV-2 than in negative samples (Figure 1B), in contrast to previous reports indicating IFN-dependent upregulation of *ACE2* expression upon SARS-CoV-2 infection [18]. Moreover, when comparing *ACE2* gene expression longitudinally in positive samples, *ACE2* expression showed a significant decrease over time, following the same trend observed with ACE2 enzymatic activity and suggesting that SARS-CoV-2 is able to downregulate the expression of its receptor.

SARS-CoV-2 viral load was quantified by qPCR at the same timepoints where ACE2 activity and gene expression were determined, to unravel putative interactions between soluble active ACE2 and SARS-CoV-2 infection. As previously reported, viral load significantly decreased from day 0 to day 3 (mean of 2 log reduction, *p*-value = 6.5 × 10^−7^, Supplementary Figure S2), showing a similar reduction to that observed for *ACE2* expression and parallel to the trend observed for ACE2 activity. No differences in viral load were identified when the data was stratified by sex (Table 1 and Supplementary Figure S2).

Interestingly, a significant positive correlation was found between ACE2 activity drop and viral load decrease over time (spearman correlation coefficient rho = 0.355, *p*-value = 0.025, Figure 1C). Indeed, *ACE2* gene expression directly correlated with viral load (spearman correlation coefficient rho = 0.352, *p*-value = 0.0259, Figure 1D), strongly suggesting a direct link between viral entry and ACE2 expression and function.

### 3.2. SARS-CoV-2 Infection Modulates Inflammatory Cytokine Expression in Upper Respiratory Track

**Figure 2:**
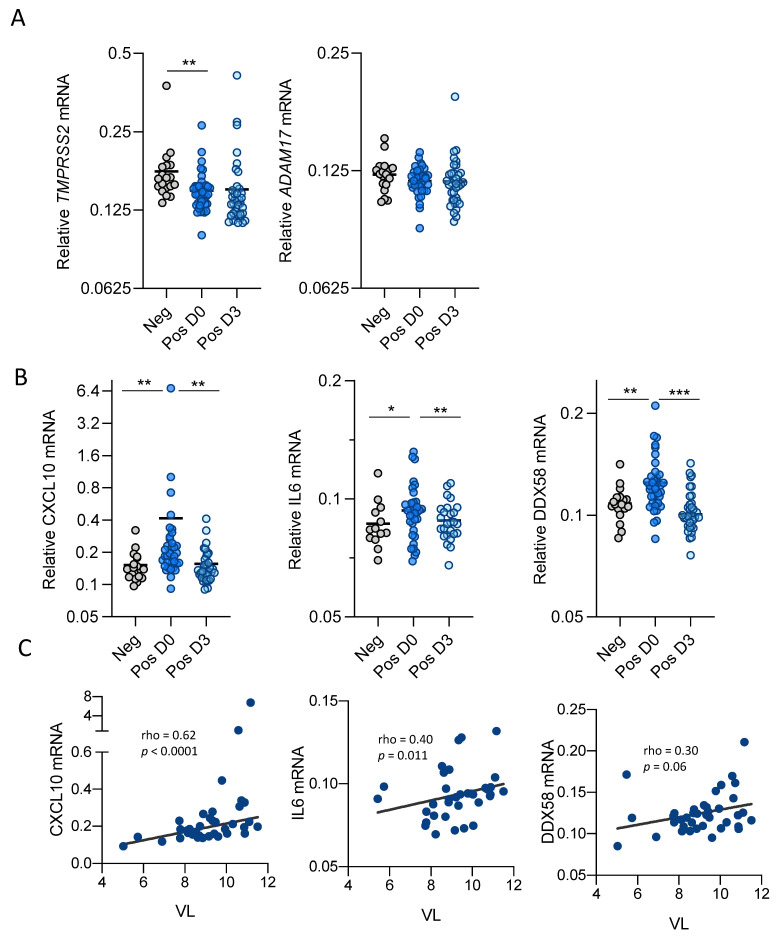
Gene expression modulation by SARS-CoV-2 in longitudinal samples of positive subjects. (**A**) Relative expression of TMPRSS2 (left) and ADAM17 (right) proteases in negative or positive SARS-CoV-2 longitudinal samples. (**B**) Relative expression of different ISGs. Relative mRNA expression was measured by quantitative PCR and normalized to GAPDH. Data represent 1/DCt individual values. (**C**) Positive correlations of DDX58, CXCL10 and IL6 gene expression with viral load. Data are shown as mean ± SEM and analyzed by Mann–Whitney U test, * *p* < 0.05, ** *p* < 0.01, *** *p* < 0.001. Linear correlation (Spearman) r and *p*-values are shown.

To further explore gene expression modulation induced by SARS-CoV-2 infection and mRNA expression of TMPRSS2 and ADAM17, the proteases able to cleave ACE2 ectodomain together with distinct ISG genes were also measured. In the case of *TMPRSS2* and *ADAM17* expression, there were no significant differences in longitudinal samples obtained at different timepoints of SARS-CoV-2 positive subjects, although *TMPRSS2* expression was lower in infected compared to uninfected subjects (Figure 2A).

On the contrary, significantly higher expression of the ISG genes, *DDX58 (RIG-I)*, *CXCL10* and *IL-6* genes was observed in positive SARS-CoV-2 samples at baseline compared to negative subjects (Figure 2B), albeit ISG expression decreased at follow-up 3 days later, suggesting that viral infection is inducing an IFN-mediated antiviral response that is rapidly counteracted by viral proteins.

Interestingly, increased expression of ISGs was associated with higher viral load (Figure 2C) but did not correlate with proteases expression (Supplementary Figure S3), also supporting the idea that SARS-CoV-2 infection is inducing an antiviral response upon viral entry that is afterwards suppressed to facilitate infection spread.

### 3.3. Soluble ACE2 Is Transiently Elevated in Plasma of SARS-CoV-2 Infected Patients and Correlates with Inflammation Markers

**Figure 3:**
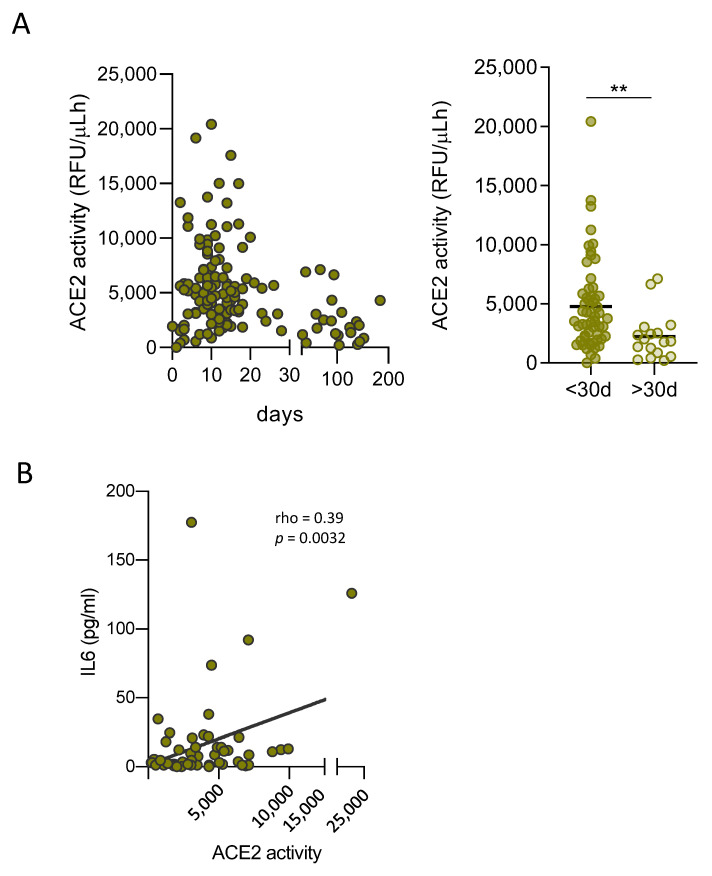
Soluble ACE2 plasma levels in COVID-19 patients. (**A**) Left, ACE2 enzymatic activity in plasma samples from SARS-CoV-2 positive subjects (*n* = 75), collected at different days from diagnosis. Right, soluble active ACE2 levels stratified by time of sampling. (**B**) Positive correlation of IL6 plasma levels and plasma ACE2 activity (*n* = 54). Data are shown as mean ± SEM and analyzed by Mann-Whitney U test, ** *p* < 0.01. Linear correlation (Spearman) r and *p*-values are shown.

To determine whether the dynamics of ACE2 function in airway epithelial cells might be indicative of systemic shedding in other tissues, ACE2 activity was measured in plasma samples from COVID-19 patients collected at different time points since diagnosis. Interestingly, we observed a significant but transient increase in soluble active ACE2 in plasma early in the first weeks of infection (Figure 3A). More importantly, soluble active ACE2 correlated with IL6 plasma levels, suggesting a direct relation between ACE2 shedding and inflammation (Figure 3B), as previously shown in other pathologies implicating endothelial cell damage [27].

## 4. Discussion

In this study, we report changes in expression of SARS-CoV-2 infection-related genes and the enzymatic activity of ACE2 in nasopharyngeal swabs and plasma samples, contributing to the understanding of the biology of early viral infection. Overall, our findings show the modulation induced by SARS-CoV-2 on its receptor, ACE2, at gene and soluble level throughout the first days after infection, which is afterwards observed in the plasma of infected patients. Moreover, we show that initial upregulation of antiviral immune response in the upper respiratory tract is directly linked to viral load, and its upregulation is rapidly reversed within days during the course of infection. However, the link between circulating ACE2 activity and inflammation is shown by the direct correlation of soluble ACE2 activity in plasma with IL-6. Our data adds key relevant knowledge to the understanding of the early antiviral host response to the SARS-CoV-2 infection at the site of viral infection, with putative implications for determining infection severity and outcome.

Soluble ACE2 has been described as a risk factor of death or cardiovascular events associated with several kidney or heart diseases [26,28,29,30], and more recently, was also reported in the general population [31]. The role of the circulating molecule has been discussed as a feedback pathway to counterbalance the RAS and hyperinflammation activation. In our study, this scenario is also plausible in the upper airway epithelium where viral entry starts to induce the inflammatory cascade that may ultimately lead to the cytokine storm [32]. Upon infection, soluble ACE2 enzymatic activity in nasopharyngeal swabs may increase in some patients, probably due to the infection itself and/or the activity of sheddase ADAM17 and TMPRSS2 [33,34]. However, longitudinal evaluation of soluble ACE2 in patient-matched samples showed a significant decrease, indicating that viral infection is significantly affecting ACE2 function. Indeed, soluble ACE2 measured in plasma showed a decrease over time, in line with previous reports [14,35,36]. Overall, we demonstrate that SARS-CoV-2 infection induces shedding of ACE2 from cell membranes, leading to increased levels of the soluble active form of ACE2 in situ; however, it is also found later on in plasma, when the infection has spread systemically.

Controversial observations have been published regarding *ACE2* gene expression in COVID-19 infections. Previous studies on SARS-CoV linked the downregulation of *ACE2* expression levels in the lungs with the pathogenicity of a viral infection as a mechanism to avoid protective levels of soluble ACE2 [20,37,38]. On the other hand, Ziegler and colleagues by analysis of single-cell RNA-seq datasets describe ACE2 as an IFN-stimulated gene in human epithelial tissues, upregulated following SARS-CoV-2 infection [18]. A recent publication reports the discovery of a truncated form of *ACE2* gene (*dACE2*) as the IFN-inducible isoform of ACE2 acting neither as a viral receptor nor as carboxypeptidase [39]. This finding would be in line with our results showing no significant changes in ACE2 soluble activity upon SARS-CoV-2 infection, concomitant to a decrease in *ACE2* gene expression. In contrast, a clear induction of IFN stimulated genes is observed upon SARS-CoV-2 infection, also supporting the idea that full length ACE2 is not an ISG and that viral infection is indeed downregulating its expression [20]. Importantly, ACE2 function correlated with viral load, further stressing the role of ACE2 as one of the key factors in SARS-CoV-2 pathogenesis. Indeed, age and sex differences in ACE2 expression have also been linked to COVID-19 susceptibility and disease outcome [40], in concordance with our data showing higher ACE2 in males compared to females [31,41].

In the setting of COVID-19 infection, soluble ACE2 has been proposed as a treatment against the spread of the virus. Clinically, changes in soluble ACE2 might imply a decrease in membrane-bound ACE2 and indicate the pathophysiological status of the patient. The presence of circulating ACE2 locally at the primary site of infection might also have implications in disease progression as a feedback pathway to counterbalance the RAS and hyperinflammation activation [42]. Parallel to the description of the biological significance of circulating ACE2, other studies showed that soluble ACE2 might be postulated as a protective molecule in some experimental models [37] or recently, as a blocker for SARS-CoV-2 virus [43]. However, further studies in extended cohorts are needed to unravel the clinical significance of soluble ACE2 needs, in particular settings and considering each clinical situation.

Although the virus-receptor interaction is needed to initiate infection, pathogenesis is a multistep process and the development of the disease is influenced by multiple factors, in which target cell factors and the capacity of the host to develop a proper immune response are key. Indeed, the fact that *TMPRSS2*, but not *ADAM17* expression was downregulated upon infection might indicate the prominent role of *TMPRSS2* in the context of SARS-CoV-2 infection. On the other hand, we observe a significant upregulation of *CXCL10*, *IL-6* and *RIG-I* expression, all playing important roles in innate immune activation and clearly indicating that SARS-CoV-2 infection induces an antiviral response characterized by ISG upregulation. Importantly, the highest levels of individual ISG expression were seen in samples with the highest viral load. However, patient-matched longitudinal specimens showed a clear reduction in ISG-induced transcription 3 days later, demonstrating that the induction of an antiviral response rapidly wanes with time. In concordance with our results, the capacity of coronaviruses to manipulate immune responses and interfere with the IFN pathway is well established, with several structural proteins (M and *n*) and non-structural proteins (NSP1 and NSP3) from SARS-CoV and MERS-CoV acting as interferon antagonists [44].

Collectively, our results support the existence of mechanisms by which SARS-CoV-2 induces shedding of ACE2, together with downregulation of *ACE2* gene expression, intracellular viral sensing and subsequent ISG induction for favoring viral replication. Deciphering the regulation of ACE2 and ISG expression and function in SARS-CoV-2 target cells is a step forward in linking ACE2 levels with viral damage and COVID-19 pathology, which may help to design better strategies to efficiently clear the SARS-CoV-2 virus and minimize tissue damage. On the other hand, understanding the innate immune responses to SARS-CoV-2 and its immunoevasion approaches will improve our understanding of pathogenesis and virus clearance, and contribute toward vaccine and immunotherapeutic design and evaluation.

## Figures and Tables

**Table 1:** Baseline characteristics of index cases.

	Swab (*n* = 40)	Plasma (*n* = 75)
**Individual’s Characteristics**		
Age (years), mean (SD)	42.1 (11.3)	51.2 (14.6)
Sex (male), *n* (%)	11 (27)	44 (58)
**Symptoms at baseline**		
Fever, N (%)	27 (67.5)	40 (53.3)
Cough, N (%)	32 (80)	49 (65.3)
Dyspnea, N (%)	6 (15)	30 (40)
**Time from onset of symptoms to sample**		
Days, median (IQR)	4 (3;6)	12 (8;18)
**LOG viral load (first sample)**		
RT-qPCR copies/mL, mean (SD)	9 (1.6)	nd

nd; not-determined

## Supplementary Materials

The following are available online at https://www.mdpi.com/article/10.3390/v13091715/s1, Figure S1: Setting up enzymatic ACE2 activity, Figure S2: Viral load quantification in SARS-CoV2 positive subjects; Figure S3: Correlation of ACE2 sheddases gene expression and SARS-CoV-2 viral load.
