# Supplementary material for: SARS-CoV-2 Infection Modulates ACE2 Function and Subsequent Inflammatory Responses in Swabs and Plasma of COVID-19 Patients
Source: Viruses. 2021 Aug 28;13(9):1715. doi: 10.3390/v13091715 (PMC8471465; doi:10.3390/v13091715)
Supplement: Supplementary file 1 [file viruses-13-01715-s001.zip › viruses-1327568-supplementary.pdf]

A

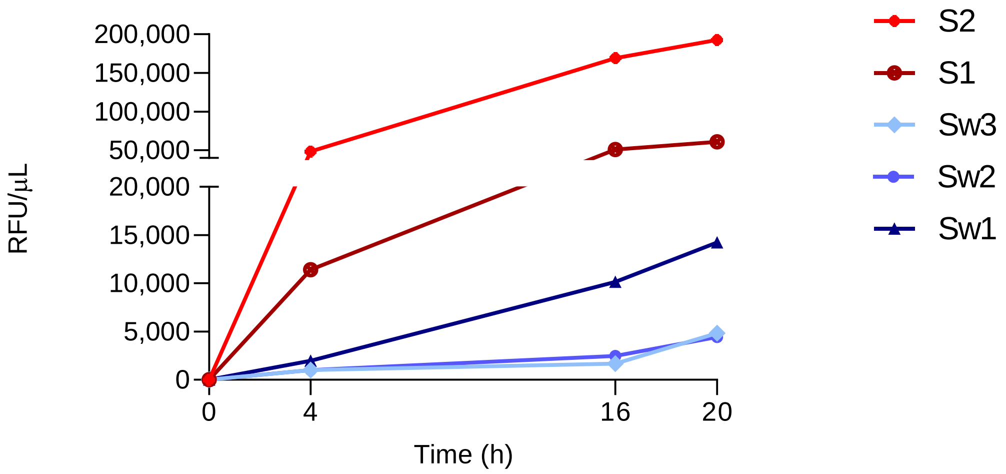

B

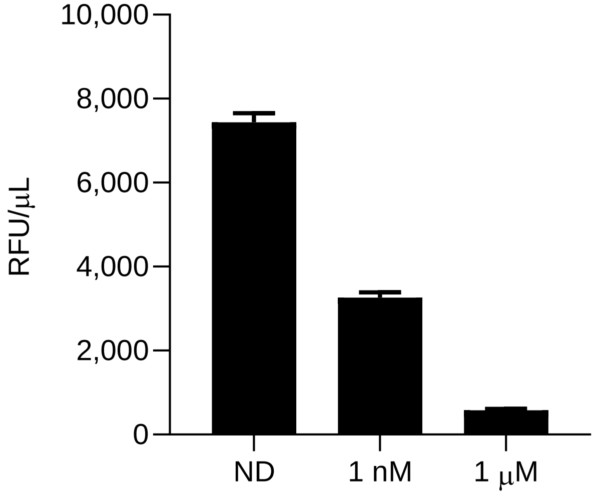

Supplementary Figure S1. Setting up enzymatic ACE2 activity. **(A)** Time course of soluble ACE2 activity in serum and nasopharyngeal swaps samples. Results are expressed as RFU per microliter of sample. Serum sample (S1 and S2); nasopharyngeal swabs (Sw1, Sw2 and Sw3) **(B)** ACE2 enzymatic activity in nasopharyngeal swabs is blocked by the specific inhibitor, MLN-4760 at 1 nM and 1 μM. ND; no drug. Results are expressed as RFU per microliter after 18h-reaction.

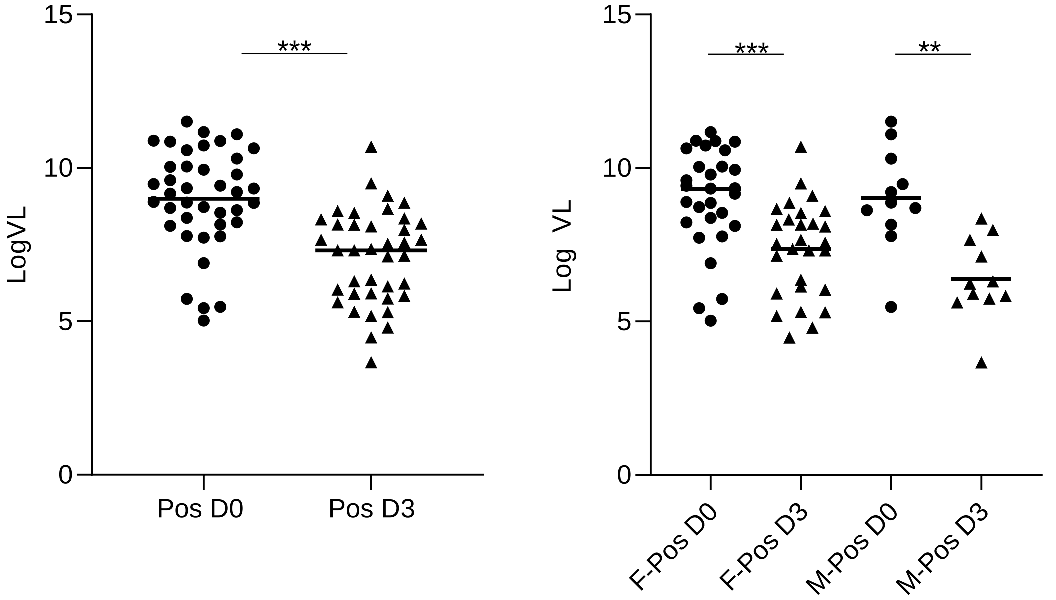

Supplementary Figure S2. Viral load in SARS-Cov-2 positive individuals. Right panel: Viral load (Log VL) at day0 and day3 of recruitment. Left panel: Sex-stratified viral load. Data are shown as mean ± SEM and analyzed by Mann-Whitney U test, $^{**}p < 0.01$, $^{***}p < 0.001$.

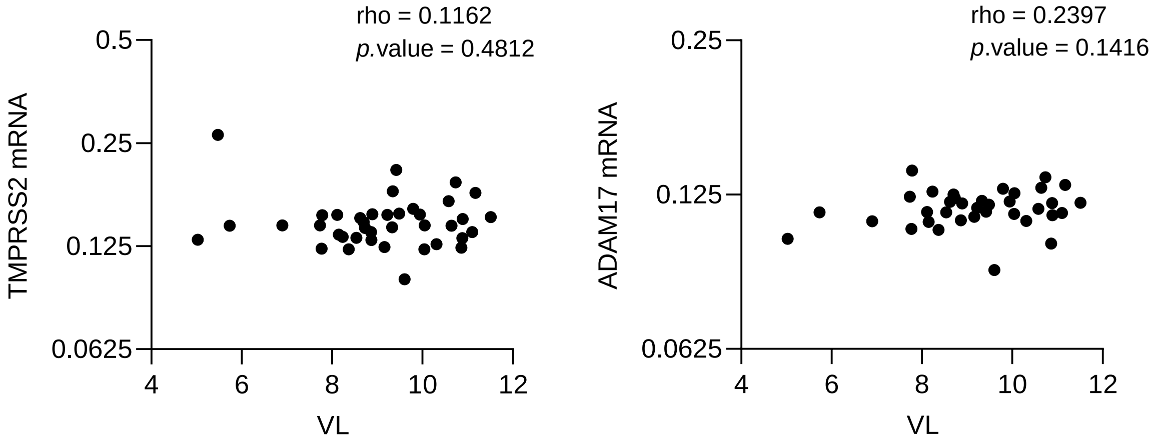

Supplementary Figure S3. Correlation of gene expression of ACE2 shedding-related enzymes and SARS-CoV-2 viral load overtime. Left panel: Correlation of TMPRSS2 gene with viral load (Log VL). Right panel: Correlation of ADAM17 gene with viral load (Log VL). Linear correlation (Spearman) rho and *p*-values are shown.
